# Influenza Pandemics and Tuberculosis Mortality in 1889 and 1918: Analysis of Historical Data from Switzerland

**DOI:** 10.1371/journal.pone.0162575

**Published:** 2016-10-05

**Authors:** Kathrin Zürcher, Marcel Zwahlen, Marie Ballif, Hans L. Rieder, Matthias Egger, Lukas Fenner

**Affiliations:** 1 Institute of Social and Preventive Medicine, University of Bern, Bern, Switzerland; 2 Swiss Tropical and Public Health Institute, Basel, Switzerland; 3 University of Basel, Basel, Switzerland; 4 Epidemiology, Biostatistics and Prevention Institute, University of Zürich, Zürich, Switzerland; New York City Department of Health and Mental Hygiene, UNITED STATES

## Abstract

**Background:**

Tuberculosis (TB) mortality declined in the northern hemisphere over the last 200 years, but peaked during the Russian (1889) and the Spanish (1918) influenza pandemics. We studied the impact of these two pandemics on TB mortality.

**Methods:**

We retrieved historic data from mortality registers for the city of Bern and countrywide for Switzerland. We used Poisson regression models to quantify the excess pulmonary TB (PTB) mortality attributable to influenza.

**Results:**

Yearly PTB mortality rates increased during both influenza pandemics. Monthly influenza and PTB mortality rates peaked during winter and early spring. In Bern, for an increase of 100 influenza deaths (per 100,000 population) monthly PTB mortality rates increased by a factor of 1.5 (95%Cl 1.4–1.6, p<0.001) during the Russian, and 3.6 (95%Cl 0.7–18.0, p = 0.13) during the Spanish pandemic. Nationally, the factor was 2.0 (95%Cl 1.8–2.2, p<0.001) and 1.5 (95%Cl 1.1–1.9, p = 0.004), respectively. We did not observe any excess cancer or extrapulmonary TB mortality (as a negative control) during the influenza pandemics.

**Conclusions:**

We demonstrate excess PTB mortality during historic influenza pandemics in Switzerland, which supports a role for influenza vaccination in PTB patients in high TB incidence countries.

## Introduction

Tuberculosis (TB) has long been a leading cause of death. Mortality data indicate that TB was epidemic in Europe for centuries, and may have peaked around 1750 in the United Kingdom and in the first half of the 19^th^ century in western continental Europe [1]. Today, TB remains a major public health problem worldwide: in 2014 there were an estimated 9.6 million new cases and 1.5 million deaths from TB [2].

Since the first recorded pandemic of presumed viral influenza in 1580, 32 influenza pandemics have been described worldwide [3]. During the 19^th^ and 20^th^ centuries, five major influenza pandemics occurred, in 1889, 1900, 1918, 1957, and 1968, and one has occurred in 2009 [3–5]. Two of these were particularly severe: the Russian pandemic in 1889 [4] and the Spanish pandemic in 1918 caused millions of deaths worldwide [6, 7]. Today, influenza is estimated to cause 3 to 5 million cases of severe illness worldwide and 250,000 to 500,000 deaths per year [8], particularly among infants, the elderly, and chronically ill people.

TB mortality steadily declined in many regions of the northern hemisphere over the past 150 years [9], but pulmonary tuberculosis (PTB) mortality peaked during the most severe global influenza pandemics, in 1918 [6, 10–14] and also in 1889 [9]. Influenza pandemics are hypothesized to have contributed to the decline of TB by killing people with TB and reducing subsequent transmission [6, 10–14]. However, information on the interaction between influenza and PTB is limited. Because TB incidence is nowadays low in affluent countries, the association between TB and influenza is difficult to study. Historic records of these major pandemics therefore offer a unique opportunity to study influenza-associated mortality in persons with PTB. Drawing upon the relevant mortality registers for the city of Bern and for Switzerland as a whole [9] we assessed the seasonality of influenza and PTB, and modeled excess PTB mortality during the 1889 (Russian) and 1918 (Spanish) influenza pandemics.

## Methods

### Data collection

We extracted historic data related to infectious disease and cancer from mortality registers and statistical reports from 1885–1950 for the city of Bern as one of the largest Swiss cities with a high TB burden [15–18], and Switzerland as a whole [19]. Mortality data stratified by month, age groups, and sex were available for the periods 1889–1894 [15–17, 20, 21] and 1919–1920 [22, 23]. Data stratified by sex were not available for the period of the Spanish influenza pandemic in Bern. To compare TB mortality trends in other regions of the northern hemisphere over a period that included one or both influenza pandemics, we also collected TB mortality data from England and Wales, 1851–1923 [24]; Germany, 1892–1950 [25]; and New York City, 1881–1950 [26, 27].

Mortality registers included deaths from ten causes confirmed by medical doctors and often determined by official autopsies [15]. We extracted causes of death due to PTB, extrapulmonary TB (EPTB), influenza, pneumonia and cancer. Influenza mortality was based on autopsy findings and clinical suspicion prior to death [17]. Pneumonia included acute respiratory diseases (acute lung abscess, acute tracheitis, and bronchitis). Cause of death due to cancer and EPTB was available for the city of Bern for both pandemics [16, 23], and for Switzerland only for the Spanish pandemic [22].

### Statistical analysis

Where necessary we rescaled historical mortality information to rates (per 100,000 population) based on census data. We restricted TB to PTB because evidence from epidemiological and experimental studies suggests an association between influenza and PTB [6, 10–13, 28], but not between influenza and EPTB. We plotted monthly and yearly mortality data to analyze mortality during and subsequent to the pandemics from 1889–1894 and 1918–1920. Age was categorized in age groups of 0–4, 5–14, 15–19, the six decade-long age groups between 20 and 79, and ≥80 years.

We used Poisson regression models adapted from previously described models [29] to evaluate excess PTB mortality rates and its association with concurrent or time-lagged mortality rates due to influenza, pneumonia, and their combination. As shown in the equation below, we modeled the log of the monthly mortality counts from PTB with a fixed offset of the log of the population count as a function of seasonal terms and mortality rates from influenza and pneumonia, and the combination of influenza and pneumonia. We modeled periods of the influenza pandemics from January 1, 1889 to December 31, 1894 (Russian pandemic), and from January 1, 1918 to December 31, 1920 (Spanish pandemic). These models allowed estimation of the factor by which the monthly PTB mortality rate increased per 100 influenza deaths per 100,000 population. We did not include a time lag between influenza and PTB mortality in the final model because the inclusion of time lags of one and two months gave a poorer model fit based on the Bayesian information criterion. The model included covariates for monthly mortality counts, time trends, and seasonal variation as follows:

$$\begin{array}{ll} \text{ln}\left({\text{Y}}_{\text{ptb}}\right)\;=\;\text{ln}\left(\text{population size}\right)+\beta_{0}+\beta_{1}\left(\text{year}\right)+{\left(\beta_{2}\left(\text{year}\right)\right)}^{2}+ & \;\left(\text{calendar time trends}\right)\\ \beta_{3}\left(\text{sin}\frac{2\pi \text{ month}}{\text{12}}\right)+\beta_{4}\left(\text{cos}\frac{2\pi \text{ month}}{\text{12}}\right)+ & \;\text{(seasonality)}\\ \beta_{5}\left(\text{influenza}\right) & \left(\text{monthly influenza mortality rate}\right) \end{array}\;$$

In addition, interactions between PTB mortality due to influenza mortality and location (Bern and Switzerland) as well as epidemic (Russian and Spanish influenza) were assessed by including interaction terms (location and epidemic) in the Poisson regression models. Finally, we also assessed models with a time lag of one month between pneumonia and PTB mortality. As a negative control, we analyzed monthly cancer and EPTB mortality to investigate potential confounding and bias in the analysis [30].

All analyses were performed in Stata version 14 (College Station, Texas, USA).

## Results

### Excess PTB mortality due to influenza

Table 1 shows the monthly relative excess PTB mortality rates per 100 deaths (per 100,000 population) due to influenza, pneumonia, and the combination of influenza and pneumonia for the two pandemics in the city of Bern and countrywide in Switzerland. For the Russian pandemic the monthly PTB mortality rate increased by a factor of 1.5 (95%Cl 1.4–1.6, p<0.001) in Bern, and by a factor of 2.0 (95%Cl 1.8–2.2, p<0.001) in Switzerland, and during the Spanish influenza by a factor of 3.6 (95%Cl 0.7–18.0, p = 0.13) in Bern and 1.5 in Switzerland (95%Cl 1.1–1.8, p = 0.004, Table 1).

**Table 1:** Relative excess pulmonary tuberculosis (PTB) mortality due to influenza, pneumonia, and combined diagnosis of influenza/pneumonia during the Russian (1889) and Spanish (1918) influenza pandemics.

Increase in PTB mortality due to:	City of Bern			Switzerland		
	Increase by a factor of n per 100 deaths^1^	95% CI	p-value	Increase by a factor of n per 100 deaths^1^	95% CI	p-value
**Russian influenza pandemic**						
Influenza	1.5	1.4–1.6	<0.001	2.00	1.8–2.2	<0.001
Pneumonia	1.5	0.9–2.3	0.12	2.4	2.1–2.7	<0.001
Influenza and pneumonia	1.4	1.1–1.8	0.005	1.50	1.4–1.6	<0.001
**Spanish influenza pandemic**						
Influenza	3.6	0.7–18.0	0.13	1.5	1.1–1.8	0.004
Pneumonia	36.4	1–1272.5	0.048	6.9	2.8–17.3	<0.001
Influenza and pneumonia	4.1	1.1–15.6	0.037	1.7	1.3–2.2	<0.001

PTB, pulmonary tuberculosis; 95% Cl, 95% confidence interval

^1^ per 100,000 population

Estimates based on the time periods between 01.01.1889 to 31.12.1894 (Russian influenza pandemic) and 01.01.1918 to 31.12.1920 (Spanish influenza pandemic)

The relative excess PTB mortality due to influenza was not significantly different between the city of Bern and Switzerland (the interaction term Bern vs. Switzerland was p = 0.45 and p = 0.79 for the Russian and the Spanish pandemic respectively). Excess PTB mortality was significantly higher during the Spanish compared to the Russian influenza pandemic for Bern (p<0.001), but higher during the Russian compared to the Spanish pandemic for Switzerland (p<0.001).

In the negative control analysis [30], there was no significant association of influenza rates with cancer mortality (S1 Table) nor with EPTB mortality (S2 Table).

### PTB mortality trends during the Russian and Spanish influenza pandemics

**Fig 1:**
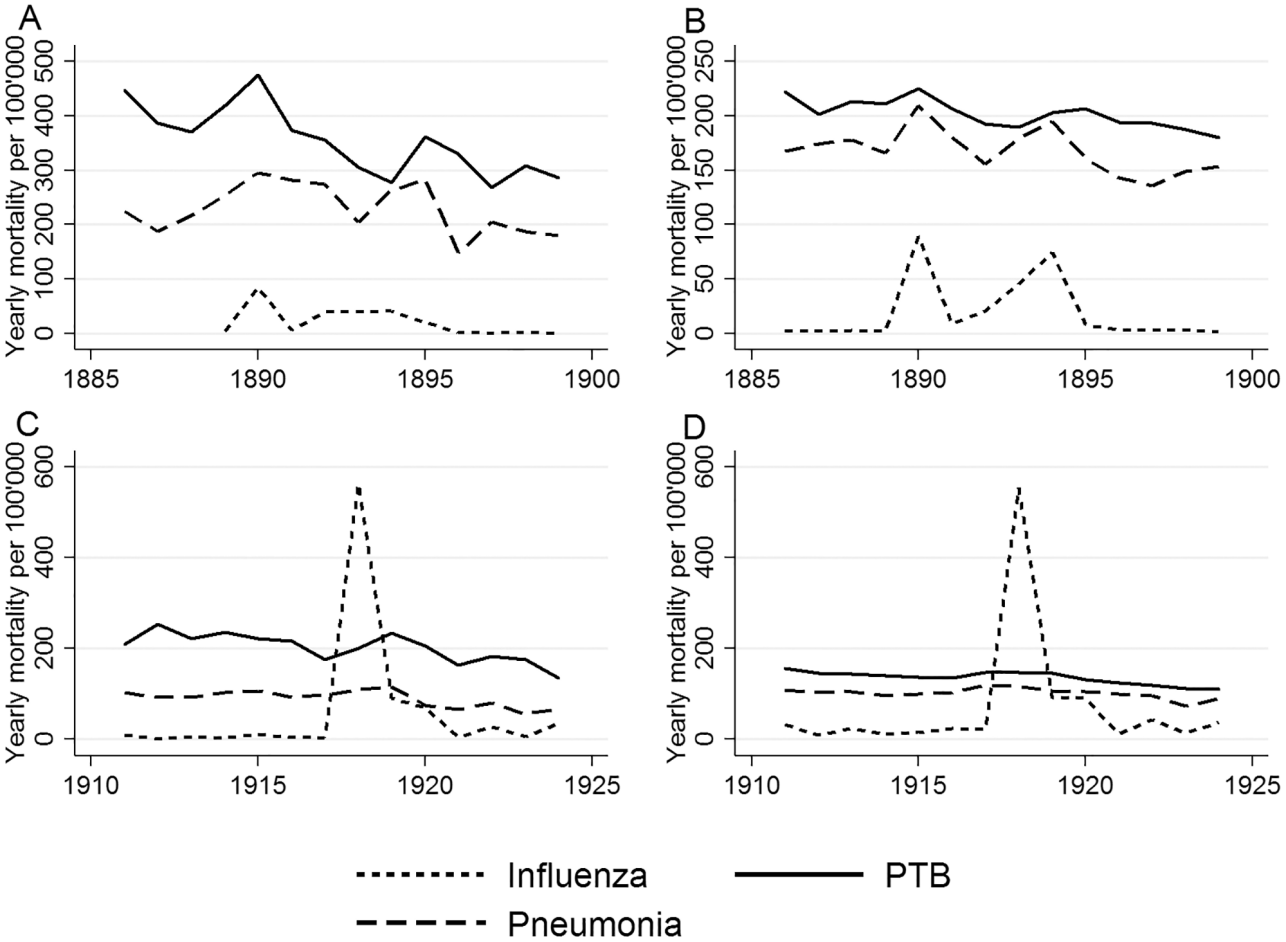
Yearly trends in pulmonary tuberculosis (PTB), influenza, and pneumonia mortality per 100,000 people during the Russian and Spanish influenza pandemics in the city of Bern (A and C) and in Switzerland (B and D).

Fig 1 shows how PTB, influenza, and pneumonia mortality rates spiked in 1889, then declined and peaked again in 1894, except for PTB mortality, which had a slightly delayed peak in 1895 in Bern and Switzerland. Both in Bern and Switzerland influenza mortality peaked again in 1918 (Fig 1). Influenza mortality in 1919 remained higher than in the years before the influenza pandemic, and there was again a small peak in 1922, while mortality due to pneumonia remained stable during these years. The peak in PTB mortality in Bern was slightly offset compared to influenza deaths.

During the 19^th^ and 20^th^ centuries, TB mortality decreased in the northern hemisphere, but peaked during the Russian (1889) and the Spanish (1918) influenza pandemics. S1 Fig reflect these trends in Bern and countrywide in Switzerland, as well as in England and Wales, Germany, and New York City.

### Seasonal patterns of TB and influenza mortality during the influenza epidemics

In temperate climates like Switzerland’s, seasonal influenza epidemics occur mainly during cold winter times [29]. Before the introduction of antibiotics against TB in the 1950s, TB mortality was highest in late winter and early spring, and lowest in late summer and autumn in the northern hemisphere [1, 31, 32], with PTB mortality usually peaking during or shortly after the influenza peak.

**Fig 2:**
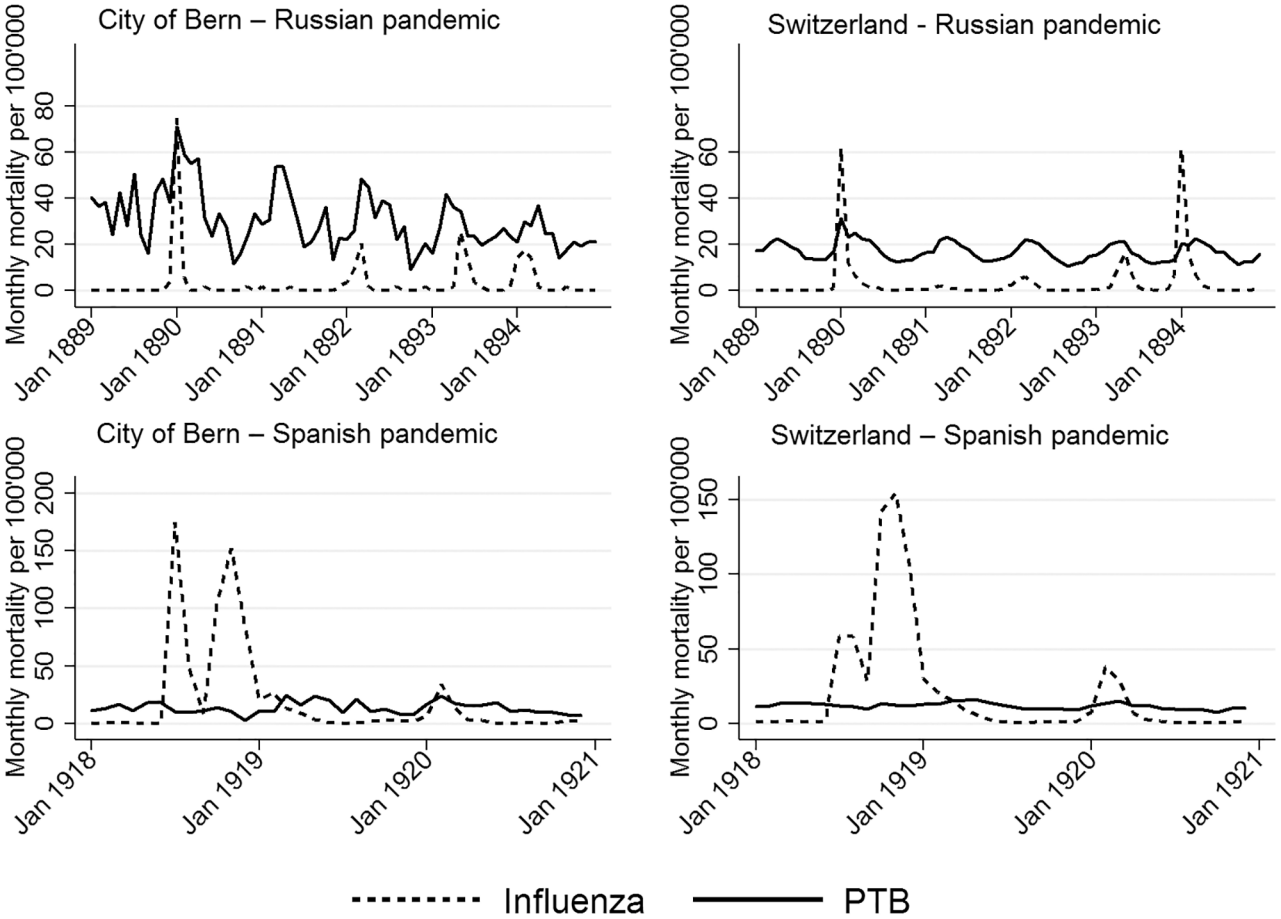
Seasonal trends in pulmonary tuberculosis (PTB) and influenza monthly mortality per 100,000 population.

With influenza, pneumonia, and PTB mortality highest during the winter months (December to April), the monthly Russian influenza mortality data in Fig 2 illustrate this seasonality in Bern and Switzerland. In both, influenza waves were observed in 1889, 1890, 1892, 1893, and 1894.

Bern and Switzerland had three influenza waves during the Spanish pandemic, the first beginning in July, the second in October/November in 1918, and a late third wave in February 1920. A further wave was observed in Bern in January 1922. PTB mortality was slightly higher during the winter months and early spring (December-April).

### Age-specific mortality of PTB and influenza

**Fig 3:**
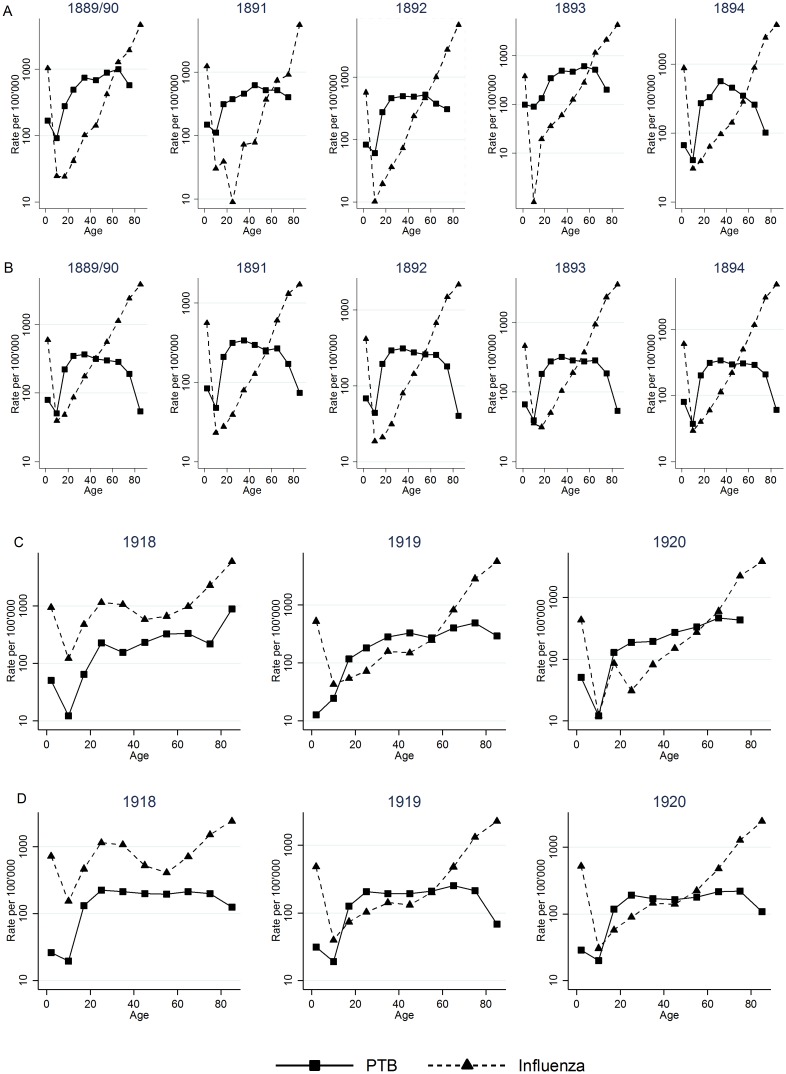
Cross-sectional mortality rates per 100,000 population of pulmonary tuberculosis (PTB) and influenza (including pneumonia and acute respiratory diseases) in Bern (A and C) and Switzerland (B and D) by age, during the influenza pandemics of 1889–1894 and 1918–1920.

The influenza mortality during the Russian pandemic was J-shaped, with high mortality among infants and the highest in older people. PTB mortality was high during infancy and early childhood (0–4 years), declined in later childhood (5–14 years), and peaked at 40–49 years (Fig 3A and 3B). PTB and influenza mortality peaked during the epidemic then declined in all age groups. During this pandemic, PTB mortality showed a familiar pattern with high mortality in infants, young adults, and elderly people, while influenza killed mostly older people.

In contrast to the earlier pandemic, the influenza mortality curves during the Spanish influenza pandemic were W-shaped (Fig 3C and 3D). In Bern and countrywide, mortality was high in infants and young adults, and rose in older people. Influenza mortality during the pandemic in 1918 was higher than PTB mortality, especially for those 20 to 40 years of age. PTB mortality that year had a shape similar to that during the Russian influenza with a peak at years 40–49, but rose again at 60–69 years.

## Discussion

PTB mortality increased during the influenza pandemics of 1889 and 1918, and declined thereafter in Bern and Switzerland. The relative excess monthly PTB mortality increased by a factor of up to two per 100 influenza deaths per 100’000 population during the Russian influenza and up to four during the Spanish influenza pandemic. The peaks in PTB mortality occurred during or slightly after the two major influenza pandemics, and were more pronounced in Bern than they were countrywide.

The observed relative excess PTB mortality due to influenza is supported by both historical and recent studies of 19^th^ and 20^th^-century influenza pandemics [6, 10–12]. Two small historical studies, one of a Swiss TB sanatorium in 1918 [10] and the other of a Danish sanatorium in 1952 [11], observed an increased risk of death among PTB patients with influenza or a worsening of the clinical course of TB due to influenza. A study from the United States suggested that selective mortality during the Spanish influenza pandemic had an effect on subsequent, underlying TB mortality [12–14]. The peak in TB mortality 1918 might also be influenced by the First World War and the beginning of the post-war recession [33]. A recent study from 1999–2009 in South Africa mirrors these findings and shows that for those both under and over 65 the relative risk of influenza-associated mortality increased [28]. However, we should also note that a study of Thai hospitalized patients presenting with acute respiratory illness did not find an increased risk of clinical worsening or mortality in PTB patients co-infected with influenza virus [34].

Seasonally, mortality for all causes of death—influenza and PTB—was highest during the winter months and early spring. Seasonal variation is also observed in other respiratory infections such as pneumonia and TB [29, 31, 35–37]. The seasonality of TB is complex, but several studies hypothesized that environmental and social factors such as temperature, humidity, poor ventilation, limited sunlight exposure and vitamin D deficiency, overcrowding, and personal contact increase PTB in winter [31, 38].

Mortality early in life influences mortality later on, so we also explored the age distribution of PTB. The age-specific influenza mortality curve for the Russian influenza is J-shaped, while the curve for the Spanish influenza is W-shaped. TB mortality, on the other hand, peaked in infants and between the ages of 20 and 40 years, and rose again in elderly people. This age-specific mortality distribution in PTB has been previously reported [1]. Most influenza pandemics have shown either a U- or J-shaped age-specific mortality curve, having disproportionately killed infants and elderly people. The W-shaped curve was previously reported in the Spanish influenza pandemic [6]. This indicates that TB and influenza mortality affected the same age groups during the Spanish influenza. In contrast, mortality was elevated among elderly people during the Russian pandemic influenza, while among younger adults many who died may have had active PTB with a severe clinical course due to subclinical influenza [6, 12–14].

All these findings suggest a causal relationship between influenza and PTB mortality that is in line with experimental studies. Infection of mice with influenza virus before or during *M*. *tuberculosis* infection significantly impaired the long-term immunological control of PTB [39], and coinfection of mice with influenza and Bacillus Calmette–Guérin (BCG) exacerbated the clinical course of the latter [40]. Furthermore, a history of PTB has been reported to be associated with both respiratory and a nonrespiratory opportunistic infection in HIV-positive patients, which suggests that functional lung impairment, may facilitate entry of respiratory pathogens [41, 42]. Taken together, these findings support the notion that influenza-associated PTB mortality may be increased by underlying lung damage caused through TB-induced chronic infection.

The main limitation of our study is the potential misclassification of the causes of death. Correctly ascertaining the cause of death, particularly in historical registers, was difficult for both influenza and TB. Because influenza was identified by clinical signs, many cases may not have been classified as influenza at the beginning of the pandemic [17, 35]. The influenza virus was first isolated and could be virologically confirmed by laboratory diagnosis only in 1933 [3, 43]. Similarly, TB diagnosis at that time was rarely bacteriologic, but rather clinical [15, 44]. Therefore, TB may have been misclassified as bacterial pneumonia subsequent to influenza or vice versa. We also recognize the underreporting in medical statistics during such large pandemics [7]. Because TB was often a chronic disease, other acute infections such as bacterial pneumonia may have been noted as cause of death instead of TB. However, the separated monthly mortality peaks of PTB and pneumonia (at least during the Russian pandemic) indicate a correct classification of the causes of death. Also, in Bern’s mortality registry death was certified by a patient’s doctor while the cause of death was determined by official autopsy, which likely reduced misclassification.

In conclusion, we observed an excess PTB mortality due to influenza during the major historic pandemics in Switzerland, and a seasonal pattern of PTB with increased PTB mortality during winter months and early spring when seasonal influenza epidemics peaked. This supports the hypothesis that influenza pandemics contributed to the historic decline of TB mortality, even before effective treatment options became available [9], by killing people with TB and reducing the pool of individuals who could potentially transmit TB [6, 12–14]. Our historical analysis also reinforces contemporary data from South Africa [28] in support of public health strategies for influenza vaccination in TB patients to prevent excess mortality in high TB incidence countries where influenza epidemics frequently occur. Influenza vaccines are not currently part of the routine vaccination among TB patients in sub-Saharan Africa and elsewhere [28], though the WHO recommended influenza vaccine for TB patients during the 2009 influenza pandemic [45]. Further clinical trials are warranted to assess the efficacy of influenza vaccination strategies on outcome and mortality of TB in high incidence countries.

## Supporting Information

S1 Fig Trends in tuberculosis (TB) mortality in different regions of the Northern Hemisphere. The dotted lines indicate the start of the respective Russian and Spanish influenza pandemics.
(TIF)

S1 File Dataset underlying the findings in the manuscript. (XLSX)

S1 Table Relative excess cancer mortality due to influenza during the Russian (1889) and Spanish (1918) influenza pandemics. Estimates based on the time periods between 01.01.1889 and 31.12.1894 (Russian influenza pandemic) and 01.01.1918 and 31.12.1920 (Spanish influenza).
(PDF)

S2 Table Relative excess extrapulmonary tuberculosis (EPTB) mortality due to influenza during the Russian and Spanish influenza pandemics. Estimates based on the periods between 01.01.1889 and 31.12.1894 (Russian influenza) and 01.01.1918 and 31.12.1920 (Spanish influenza).
(PDF)
