# Supplementary figures and images for: Influenza Pandemics and Tuberculosis Mortality in 1889 and 1918: Analysis of Historical Data from Switzerland
Source: PLoS One. 2016 Oct 5;11(10):e0162575. doi: 10.1371/journal.pone.0162575 (PMC5051959; doi:10.1371/journal.pone.0162575)

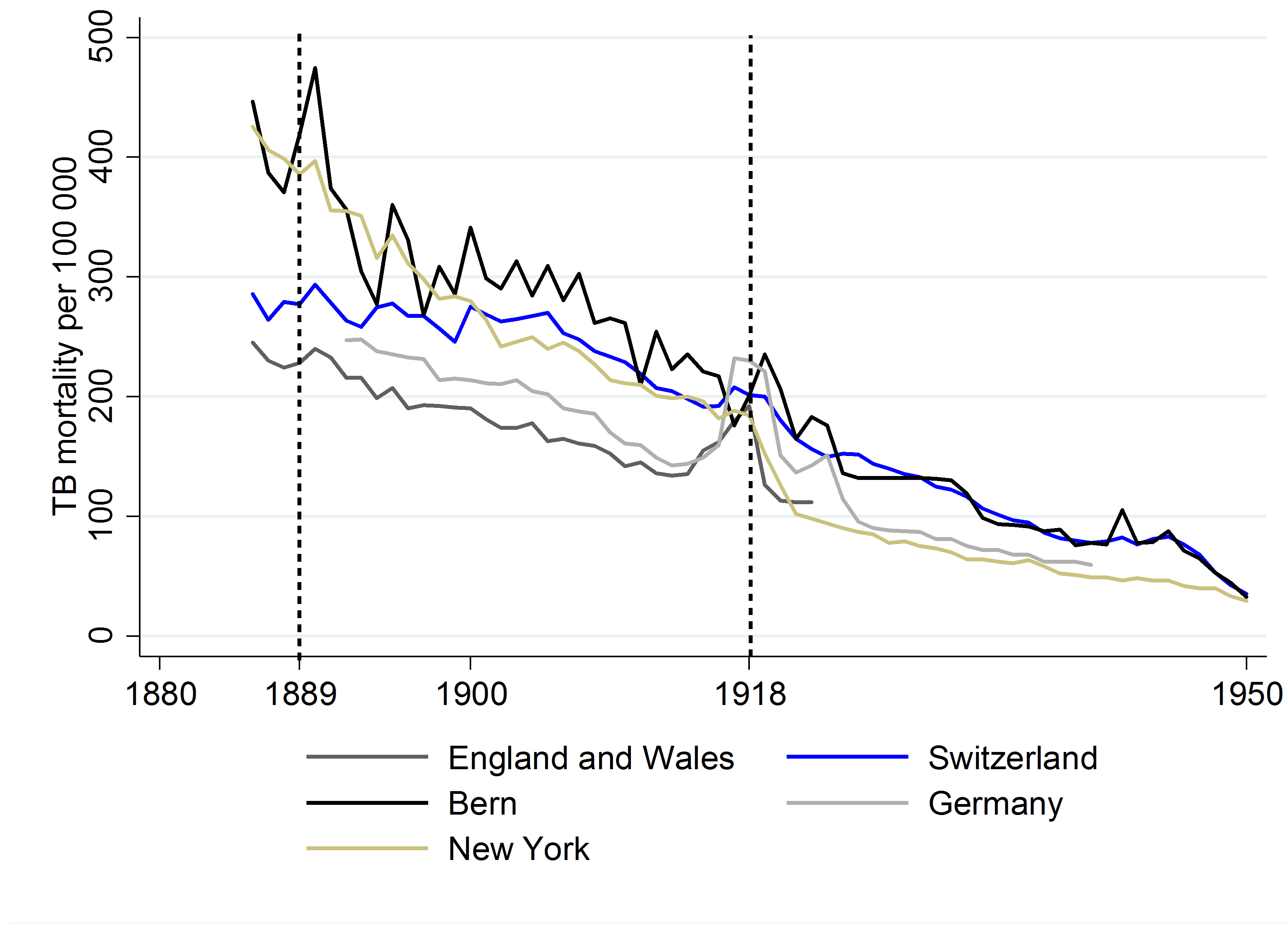

Supplement: S1 Fig — The dotted lines indicate the start of the respective Russian and Spanish influenza pandemics. (TIF) [file pone.0162575.s001.tif]
