# Supplementary material for: Influenza Pandemics and Tuberculosis Mortality in 1889 and 1918: Analysis of Historical Data from Switzerland
Source: PLoS One. 2016 Oct 5;11(10):e0162575. doi: 10.1371/journal.pone.0162575 (PMC5051959; doi:10.1371/journal.pone.0162575)
Supplement: S1 Table — Estimates based on the time periods between 01.01.1889 and 31.12.1894 (Russian influenza pandemic) and 01.01.1918 and 31.12.1920 (Spanish influenza). (PDF) [file pone.0162575.s003.pdf]

Table S1. Relative excess cancer mortality due to influenza during the Russian (1889) and Spanish (1918) influenza pandemics.

Increase in cancer mortality due to:	City of Bern			Switzerland		
	Increase by a factor of n per 100 deaths[1]	95% CI	p-value	Increase by a factor of n per 100 deaths[1]	95% CI	p-value
**Russian influenza pandemic**						
Influenza	0.7	0.3-1.8	0.5	NA	NA	NA
**Spanish influenza pandemic**						
Influenza	0.3	0.03-3.2	0.3	1	0.8-1.4	0.8

95%CI, 95% confidence interval; NA, not available

[1] per 100,000 population

Estimates based on the time periods between 01.01.1889 and 31.12.1894 (Russian influenza pandemic) and 01.01.1918 and 31.12.1920 (Spanish influenza).
