# Supplementary material for: Influenza Pandemics and Tuberculosis Mortality in 1889 and 1918: Analysis of Historical Data from Switzerland
Source: PLoS One. 2016 Oct 5;11(10):e0162575. doi: 10.1371/journal.pone.0162575 (PMC5051959; doi:10.1371/journal.pone.0162575)
Supplement: S2 Table — Estimates based on the periods between 01.01.1889 and 31.12.1894 (Russian influenza) and 01.01.1918 and 31.12.1920 (Spanish influenza). (PDF) [file pone.0162575.s004.pdf]

Table S2. Relative excess extrapulmonary tuberculosis mortality due to influenza during the Russian and Spanish influenza pandemics.

Increase in EPTB mortality due to:	City of Bern			Switzerland		
	Increase by a factor of n per 100 deaths[1]	95% CI	p-value	Increase by a factor of n per 100 deaths[1]	95% CI	p-value
**Russian influenza pandemic**						
Influenza	0.5	0.1-1.7	0.3	NA	NA	NA
**Spanish influenza pandemic**						
Influenza	0.5	0.02-11.4	0.7	0.7	0.5-1.1	0.13

95%CI, 95% confidence interval; EPTB, extrapulmonary tuberculosis; NA, not available

[1] per 100,000 population

Estimates based on the periods between 01.01.1889 and 31.12.1894 (Russian influenza) and 01.01.1918 and 31.12.1920 (Spanish influenza).
